# Greenness, Genetic Predisposition, and Tinnitus

**DOI:** 10.1002/advs.202306706

**Published:** 2024-03-06

**Authors:** Lan‐Lai Yuan, Dan‐Kang Li, Yao‐Hua Tian, Yu Sun

**Affiliations:** ^1^ Department of Otorhinolaryngology, Union Hospital, Tongji Medical College Huazhong University of Science and Technology Wuhan 430022 China; ^2^ Ministry of Education Key Laboratory of Environment and Health and State Key Laboratory of Environmental Health (Incubating) School of Public Health Tongji Medical College Huazhong University of Science and Technology Wuhan 430030 China; ^3^ Department of Maternal and Child Health, School of Public Health, Tongji Medical College Huazhong University of Science and Technology Wuhan 430030 China; ^4^ Institute of Otorhinolaryngology, Union Hospital, Tongji Medical College Huazhong University of Science and Technology Wuhan 430022 China; ^5^ Hubei Province Key Laboratory of Oral and Maxillofacial Development and Regeneration Wuhan 430022 China

**Keywords:** genetic predisposition, residential greenness, tinnitus, UK biobank

## Abstract

This study aimed to investigate the association between residential greenness and tinnitus and the potential interaction between greenness and genetic predisposition to tinnitus. The normalized difference vegetation index (NDVI) is used to measure residential greenness. The tinnitus is defined based on self‐reported. In the cross‐sectional analyses, logistic regression models are used for the baseline sample of the United Kingdom Biobank cohort. In the secondary analysis, a Cox proportional hazard model is used for a subsample of participants who completed the tinnitus questionnaire at follow‐up. In the cross‐sectional analysis including 106471 participants, higher residential greenness is associated with lower odds of tinnitus for each interquartile range increase in continuous NDVI, with an adjusted odds ratio of 0.97 (95% confidence interval: 0.95 to 0.99) for tinnitus. A similar association is observed in the longitudinal analysis, with an adjusted hazard ratio of 0.92 (95% confidence interval: 0.86 to 0.98) for the association of NDVI increased per interquartile range with incident tinnitus. Moreover, there is a significant interaction between greenness and genetic predisposition to tinnitus (*P* < 0.05). This study suggested that residential greenness is negatively associated with tinnitus. Greenness and genetic predisposition to tinnitus are found to have a significant interaction.

## Graphical abstract

The association between residential greenness and tinnitus has not been investigated. In this large population‐based study, higher residential greenness is associated with a reduced risk of tinnitus. There is a significant interaction between greenness and genetic predisposition to tinnitus. To the best of the knowledge, this is the first study to investigate the association between greenness and tinnitus.

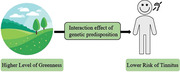

## 1 Introduction

Tinnitus is a common condition defined as the abnormal perception of sound in the head or ears without any external acoustical source.^[^ ^1^, ^2^ ^]^ Tinnitus is usually categorized into two main types: objective and subjective, with the latter being the most common.^[^ ^1^, ^2^ ^]^ The prevalence rate of tinnitus in adults reported a range of 10%–25%, depending on the different definitions in the studies.^[^ ^2^ ^]^ Most people experience transient tinnitus at some point with barely any effect, whereas some people are severely bothered by it. The bothersome tinnitus has a serious impact on the quality of life of these patients, who often experience sleep disturbances, poor concentration, frequent mood swings, etc.^[^ ^2^, ^3^ ^]^ In some cases, tinnitus may be related to psychological conditions such as depression and anxiety.^[^ ^4^, ^5^ ^]^ Additionally, the economic consequences of tinnitus are remarkable. It is estimated that the average annual cost of tinnitus treatment per patient in the United Kingdom (UK) is 717 pounds, comparable to the annual healthcare bill of 750 million pounds for the National Health Service.^[^ ^6^ ^]^ In short, tinnitus can be described as a burden on both personal and social levels.^[^ ^7^ ^]^ The causes of tinnitus are complex and related to multiple factors, among which hearing loss^[^ ^8^, ^9^ ^]^ and stress^[^ ^10^, ^11^ ^]^ are important risk factors for tinnitus. Many studies have also identified other risk factors, including ear diseases (e.g., otitis media and Meniere's disease), noise exposure, ototoxic drugs, head trauma, smoking, alcohol intake, obesity, metabolic diseases (e.g., hyperlipidemia and diabetes), cardiovascular diseases (e.g., hypertension), and neurological disorders (e.g., meningitis).^[^ ^1^, ^8^ ^]^

The primary driver of tinnitus has been attributed to environmental factors (as opposed to genetic factors) that largely affect its onset and severity.^[^ ^12^, ^13^ ^]^ Over the past decades, however, increasing evidence has supported the heritability of tinnitus.^[^ ^12^ ^]^ The heritability of tinnitus was between 0.27 and 0.56.^[^ ^14^, ^15^, ^16^ ^]^ This increased to 0.68 for men with bilateral tinnitus when gender was considered.^[^ ^15^ ^]^ Furthermore, in 2020, a genome‐wide association study (GWAS)^[^ ^17^ ^]^ using UK Biobank cohort data identified six significant loci for tinnitus. Two single nucleotide polymorphisms (SNPs) in the methionine sulfoxide reductase A (MSRA, rs11249981) and collagen type XI alpha 1 chain (COL11A1, rs143424888) genes were independently linked to tinnitus.^[^ ^17^ ^]^ The MSRA gene on chromosome 8p23.1 encodes an enzyme (MsrA) that performs the enzymatic reduction of methionine sulfoxide to methionine,^[^ ^18^ ^]^ helping to repair oxidatively inactivated protein,^[^ ^18^, ^19^ ^]^ and remove reactive oxygen species through the cyclic oxidation/reduction mechanism.^[^ ^20^ ^]^ The gene is highly expressed in brain tissue and has been reported to be associated with neuroticism and mental health (e.g., depression).^[^ ^18^, ^21^, ^22^ ^]^

The beneficial effects of green spaces on various health outcomes are gaining attention. However, there is no research on the potential benefits of green spaces for tinnitus. Of note, the beneficial effects of green spaces on tinnitus are possible. First, consistent evidence from different studies^[^ ^23^, ^24^ ^]^ has indicated that green spaces, as a potentially powerful natural resource, can help reduce stress, which may be beneficial for tinnitus.^[^ ^10^, ^11^ ^]^ Second, green spaces can provide natural sounds such as birdsong and the wind through the trees.^[^ ^25^, ^26^ ^]^ The perception of tinnitus may be lessened by listening to such sounds.^[^ ^1^, ^27^ ^]^ At the same time, the sounds of nature can facilitate stress recovery by inducing positive emotions.^[^ ^25^, ^28^ ^]^ Lastly, a series of research has indicated that greenness is associated with a lower likelihood of obesity,^[^ ^29^ ^]^ cardiovascular diseases,^[^ ^30^ ^]^ and metabolic disorders,^[^ ^31^ ^]^ which are linked to a higher risk of tinnitus.^[^ ^1^, ^8^ ^]^

Although a variety of treatments are available for tinnitus, there is no effective treatment option for tinnitus due to its clinical heterogeneity, namely that the acoustic characteristics, influencing factors, and underlying pathophysiology can vary.^[^ ^32^ ^]^ Therefore, it is important to understand the factors that may contribute to tinnitus relief, which can help develop effective and applicable prevention and intervention strategies. Based on the hypothesis of the potentially beneficial effects of green spaces on tinnitus, this study aimed to investigate whether residential greenness exposure was negatively associated with tinnitus using a large cross‐sectional sample from the UK Biobank. Given the distinct genetic predisposition of individuals with tinnitus, we also investigated whether greenness had an interaction effect with genetic predisposition to tinnitus. As a secondary analysis, we performed a longitudinal analysis on a subsample of those who completed a tinnitus questionnaire during follow‐up to replicate the negative association of greenness with tinnitus.

## 2 Results

### 2.1 Characteristics of Participants in the Primary Analysis

Among a total of 106471 participants included in the primary analysis, the median age [(interquartile range (IQR)] was 58.0 (13.0) years, with a range of 40–72 years, 53.9% (n = 57362) were female, and the majority of participants (94.1%, n = 100155) were white (**Table** 1). 17.3% (n = 18418) of participants in the analyzed sample reported the current tinnitus. The median (IQR) of the normalized difference vegetation index (NDVI) was 0.21 (0.19), ranging from −0.50 to 0.70. ​Participants who reported current tinnitus were more likely to be male, older, less educated, and in the fourth quartile of the Townsend deprivation index (Table 1).

**Table 1:** Characteristics of participants in the primary analysis.

	Current of tinnitus			*P* ^a)^
	Total (n = 106471)	No (n = 88053)	Yes (n = 18418)	
Age (years), median (IQR)	58.0 (13.0)	58.0 (13.0)	61.0 (11.0)	<0.001
NDVI, median (IQR)	0.21 (0.19)	0.21 (0.19)	0.21 (0.19)	<0.001
Sex (%)				<0.001
Female	57362 (53.9)	48981 (55.6)	8381 (45.5)	
Male	49109 (46.1)	39072 (44.4)	10037 (54.5)	
Race (%)				<0.001
White	100155 (94.1)	82593 (93.8)	17562 (95.4)	
Non‐white	6316 (5.9)	5460 (6.2)	856 (4.6)	
Qualifications (%)				<0.001
College or university degree	37543 (35.3)	31984 (36.3)	5559 (30.2)	
A levels or AS levels or equivalent	12389 (11.6)	10430 (11.8)	1959 (10.6)	
O levels, GCSEs, or CSEs or equivalent	28827 (27.1)	24023 (27.3)	4804 (26.1)	
NVQ, HND, HNC, or other professional qualification	12623 (11.9)	10067 (11.4)	2556 (13.9)	
None of the above	15089 (14.2)	11549 (13.1)	3540 (19.2)	
Employment (%)				<0.001
Employed	60049 (56.4)	51332 (58.3)	8717 (47.3)	
Retired	37090 (34.8)	29251 (33.2)	7839 (42.6)	
Other	9332 (8.8)	7470 (8.5)	1862 (10.1)	
Townsend deprivation index (%)				<0.001
1st (least deprived)	26599 (25.0)	22115 (25.1)	4484 (24.3)	
2nd	26627 (25.0)	21924 (24.9)	4703 (25.5)	
3rd	26626 (25.0)	22232 (25.2)	4394 (23.9)	
4th (most deprived)	26619 (25.0)	21782 (24.7)	4837 (26.3)	
Drink frequency (%)				<0.001
Daily or almost daily	22920 (21.5)	18921 (21.5)	3999 (21.7)	
Three or four times a week	24849 (23.3)	20809 (23.6)	4040 (21.9)	
Once or twice a week	27036 (25.4)	22411 (25.5)	4625 (25.1)	
One to three times a month	11841 (11.1)	9795 (11.1)	2046 (11.1)	
Special occasions only	11828 (11.1)	9650 (11.0)	2178 (11.8)	
Never	7997 (7.5)	6467 (7.3)	1530 (8.3)	
Smoking (%)				<0.001
Never	58910 (55.3)	49428 (56.1)	9482 (51.5)	
Previous	37326 (35.1)	30127 (34.2)	7199 (39.1)	
Current	10235 (9.6)	8498 (9.7)	1737 (9.4)	
BMI, kg m^−^^2^ (%)				<0.001
<25	36402 (34.2)	30652 (34.8)	5750 (31.2)	
≥25 and <30	45204 (42.5)	37134 (42.2)	8070 (43.8)	
≥30	24865 (23.4)	20267 (23.0)	4598 (25.0)	
Diabetes (%)				<0.001
No	100995 (94.9)	83684 (95.0)	17311 (94.0)	
Yes	5476 (5.1)	4369 (5.0)	1107 (6.0)	
Vascular problems (%)				<0.001
None	77415 (72.7)	64992 (73.8)	12423 (67.5)	
Hypertension	23580 (22.1)	18957 (21.5)	4623 (25.1)	
Heart attack, angina, or stroke	2670 (2.5)	2045 (2.3)	625 (3.4)	
Hypertension, and heart attack, angina, or stroke	2806 (2.6)	2059 (2.3)	747 (4.1)	
Hearing difficulty (%)				<0.001
No	77360 (72.7)	69317 (78.7)	8043 (43.7)	
Yes	29111 (27.3)	18736 (21.3)	10375 (56.3)	
Hearing aid use (%)				<0.001
No	103247 (97.0)	86336 (98.1)	16911 (91.8)	
Yes	3224 (3.0)	1717 (1.9)	1507 (8.2)	
Work noise exposure (%)				<0.001
No	82491 (77.5)	70352 (79.9)	12139 (65.9)	
Yes	23980 (22.5)	17701 (20.1)	6279 (34.1)	
Music noise exposure (%)				<0.001
No	93698 (88.0)	78267 (88.9)	15431 (83.8)	
Yes	12773 (12.0)	9786 (11.1)	2987 (16.2)	

IQR, interquartile range; NDVI, normalized difference vegetation index; GCSEs, general certificate of secondary educations; CSEs, certificate of secondary educations; NVQ, national vocational qualification; HND, higher national diploma; HNC, higher national certificate; BMI, body mass index.

Data are shown as means (standard deviation) or medians (IQR) for continuous variables and numbers (percentages) for categorical variables.

^a)^ Significant differences (*P* < 0.05) were calculated using the Wilcoxon rank sum test for continuous variables and the Chi‐square test for categorical variables.

### 2.2 Cross‐Sectional Association between Residential Greenness and Tinnitus

**Table** 2 shows a statistically significant association between residential greenness and tinnitus. Higher residential greenness exposure was associated with lower odds of tinnitus for each IQR increase in continuous NDVI (Table 2), with an adjusted odds ratio (OR) of 0.97 [95% confidence interval (95% CI): 0.95 to 0.99] for tinnitus. We observed similar results in the five‐factor NDVI [compared with NDVI in the first quintile, the adjusted ORs (95% CI) for tinnitus in the second, third, fourth, and fifth quintiles of NDVI were 0.97 (95% CI: 0.92 to 1.02; *P* = 0.252), 0.95 (95% CI: 0.90 to 1.00; *P* = 0.052), 0.95 (95% CI: 0.90 to 1.00; *P* = 0.037), and 0.95 (95% CI: 0.90 to 0.99; *P* = 0.029), respectively], the *P*‐trend for the association of greenness with tinnitus within quintiles of the NDVI was 0.018. In addition, the restricted cubic spline model showed a linear association between NDVI and the odds of tinnitus (*P* for nonlinear association = 0.228, Figure S1, Supporting Information).

**Table 2:** Cross‐sectional association between residential greenness and the odds of tinnitus (n = 106471).

	Unadjusted OR (95% CI)	*P*	Adjusted^a)^ OR (95% CI)	*P*
NDVI, per IQR	0.97 (0.96,0.99)	< 0.001	0.97 (0.95,0.99)	0.003
NDVI‐quintile				
Q1	1.00 (ref)		1.00 (ref)	
Q2	0.98 (0.93,1.03)	0.364	0.97 (0.92,1.02)	0.252
Q3	0.94 (0.90,0.99)	0.020	0.95 (0.90,1.00)	0.052
Q4	0.91 (0.86,0.95)	< 0.001	0.95 (0.90,1.00)	0.037
Q5	0.95 (0.90,1.00)	0.036	0.95 (0.90,0.99)	0.029
*P*‐trend^b)^		0.001		0.018

OR, odds ratio; CI, confidence interval; NDVI, normalized difference vegetation index; IQR, interquartile range; Q1, Q2, Q3, Q4 and Q5: 1st (−0.4964 – 0.0816, n = 21291), 2nd (0.0817 – 0.1848, n = 21278), 3rd (0.1849 – 0.2417, n = 21286), 4th (0.2418 – 0.3253, n = 21303), and 5th (0.3254 – 0.7032, n = 21313) quintiles of NDVI respectively.

The NDVI of each IQR is equivalent to 0.19.

^a)^ Adjusted for age, sex, race, Townsend deprivation index, qualifications, and employment;

^b)^ *P‐*trend describes the association between greenness and tinnitus within quintiles for NDVI.

### 2.3 Cross‐Sectional Association between Residential Greenness and Tinnitus Stratified by the Genotypes of rs11249981 and rs143424888

**Figure 1:**
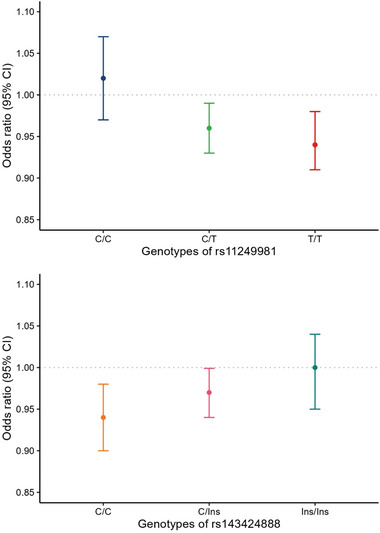
Cross‐sectional association between residential greenness and the odds of tinnitus stratified by the genotypes of rs11249981 and rs143424888 among 106471 participants.

The association between residential greenness and tinnitus stratified by the genotypes of rs11249981 and rs143424888 is shown in **Figure** **1** and Table S1 (Supporting Information). We observed that the negative association between greenness and tinnitus was stronger with the increasing rs11249981 T allele count (Figure 1; Tables S1 and S2, Supporting Information). The adjusted OR for each IQR increase in NDVI was 0.94 (95% CI: 0.91 to 0.98) in participants with the homozygous T genotype. The interaction of the MSRA gene polymorphism with NDVI showed statistical significance (*P* for interaction = 0.024). Furthermore, the OR for the association of greenness with tinnitus was lowest in the group with the homozygous C genotype of rs143424888 (Figure 1; Table S1, Supporting Information), with a significant interaction (*P* for interaction = 0.042).

CI, confidence intervals. Note: Ins represents the insertion of CACGTGATCT. Odds ratios and corresponding 95% CIs were calculated based on each interquartile range (IQR) change in NDVI, adjusted for age, sex, race, Townsend deprivation index, qualifications, and employment.

Interaction *P*‐values of rs11249981 and rs143424888 on the relationship between residential greenness and tinnitus were 0.024 and 0.042, respectively.

### 2.4 Additional Analysis and Sensitivity Analyses for the Primary Analysis

In the mediation analysis (Table S3, Supporting Information), we observed that residential greenness was indirectly negatively associated with tinnitus through self‐reported hearing difficulty (*β* = −0.009; 95% CI: −0.013 to −0.005), showing that self‐reported hearing difficulty significantly mediated 37.5% of the association. The mediating effects of body mass index and vascular problems were tiny, while it was not observed for diabetes.​

Sensitivity analysis suggested a significant interaction between greenness and polygenic risk score (PRS) for tinnitus (*P* for interaction = 0.004). The results of the joint association of greenness and PRS with tinnitus were more significant in participants with low PRS (Table S4, Supporting Information). In addition, PRS for tinnitus was associated with an increased risk of tinnitus (Table S5, Supporting Information). Among the 99689 participants included in the PRS analysis, the association of NDVI with tinnitus remained significant (Table S6, Supporting Information), and the interactions of rs11249981 and rs143424888 with greenness were still observed (Table S7, Supporting Information). There was no significant change in the results after the household income was adjusted further (Table S8, Supporting Information). When the main model with additional adjustments for smoking, drinking frequency, occupational and musical noise exposure, and all of the above, we observed no significant change in results (Table S9, Supporting Information). The results remained unchanged after excluding participants who had used hearing aids and had lived in their current residence for less than three years (Table S10, Supporting Information). No effect modification of comorbidities was observed (Table S11, Supporting Information). Similar results were observed using the alternative definition of tinnitus (Table S12, Supporting Information).

### 2.5 Secondary Analysis

**Table** 3 presents the association of greenness at baseline with incident tinnitus. A negative association was observed between NDVI and incident tinnitus, with an estimated hazard ratio (HR) of 0.92 (95% CI: 0.86 to 0.98). Compared to the first quintile of NDVI, the fourth and fifth quintiles of NDVI were associated with a lower risk of tinnitus, with HRs of 0.73 (95% CI: 0.61 to 0.87) for the fourth quintile and 0.85 (95% CI: 0.73 to 0.99) for the fifth quintile (*P*‐trend < 0.001 within quintiles for NDVI).

**Table 3:** Prospective association between residential greenness at baseline and the hazard ratio of incident tinnitus among 15607 participants.

	Case/Total	Adjusted^a)^ HR (95% CI)	*P*
NDVI, per IQR	1438/15607	0.92 (0.86, 0.98)	0.009
NDVI‐quintile			
Q1	308/3072	1.00 (ref)	
Q2	274/2832	1.03 (0.87, 1.21)	0.733
Q3	294/3354	0.99 (0.84, 1.16)	0.896
Q4	224/3041	0.73 (0.61, 0.87)	<0.001
Q5	338/3308	0.85 (0.73, 0.99)	0.036
*P*‐trend^b)^			<0.001

HR, hazard ratio; CI, confidence interval; NDVI, normalized difference vegetation index; IQR, interquartile range; Q1, Q2, Q3, Q4 and Q5: 1st (−0.41 – 0.08), 2nd (0.09 – 0.17), 3rd (0.18 – 0.22), 4th (0.23 – 0.31), and 5th (0.32 – 0.70) quintiles of NDVI respectively.

The NDVI of each IQR is equivalent to 0.18.

^a)^ Adjusted for age, sex, race, Townsend deprivation index, qualifications, and employment.

^b)^ *P*‐trend describes the association between greenness and tinnitus within quintiles for NDVI.

## 3 Discussion

In this large study, based on a cross‐sectional sample of 106471 participants in the UK Biobank cohort, we found that residential greenness was associated with a lower risk of tinnitus. This association was replicated in a longitudinal analysis conducted as the secondary analysis. In addition, a significant interaction was observed between greenness and genetic predisposition to tinnitus (including the MSRA and COL11A1 gene polymorphisms, and PRS for tinnitus). We also found that residential greenness might indirectly influence tinnitus by reducing the risk of self‐reported hearing difficulty. To our knowledge, this was the first study to report an association between residential greenness and a lower likelihood of tinnitus. This study may have vital instructive significance, such as developing cost‐effective environmental greenness strategies for preventing or treating tinnitus and identifying those who may benefit most from them.

Although the potential mechanisms for the health benefits of residential greenness are unclear, several possible mechanisms have been suggested,^[^ ^26^ ^]^ like encouraging physical activity, reducing exposure to harm (e.g., air pollution and heat), facilitating social cohesion, and relieving stress. One or more of these mechanisms might also be involved in the association between residential greenness and tinnitus. Notably, stress reduction could be a potentially applicable explanatory pathway. Some studies have shown that stress is associated with tinnitus.^[^ ^10^, ^11^ ^]^ Furthermore, the limbic system, a key region to regulate the stress response, was activated in the presence of tinnitus, which provided a neuroanatomical correlation to stress‐related tinnitus.^[^ ^33^, ^34^ ^]^ However, greenness might help relieve mental and physical stress by promoting physical activity,^[^ ^24^ ^]^ inducing positive emotions,^[^ ^23^ ^]^ and reducing physiological stress markers such as salivary cortisol,^[^ ^35^ ^]^ thereby benefiting tinnitus. Additionally, natural sounds from green spaces (e.g., birdsong, the wind through the trees, and water sounds) could not only induce a positive emotional response to alleviate stress,^[^ ^27^, ^28^ ^]^ but might also reduce the perceived burden and intensity of tinnitus by means of the general mechanisms of sound therapy – masking and distraction.^[^ ^27^, ^36^, ^37^, ^38^ ^]^

The pathophysiology of tinnitus is complicated, involving non‐auditory networks and auditory pathways.^[^ ^39^ ^]^ Apart from the limbic system being proposed as possibly playing a role in the regulation or maintenance of tinnitus,^[^ ^34^ ^]^ tinnitus has also been regarded as a neuroplastic response to hearing loss (sensory deprivation),^[^ ^8^, ^40^ ^]^ leading to increased spontaneous activity and synchronization.^[^ ^41^ ^]^ Hearing damage can often trigger tinnitus.^[^ ^42^ ^]^ When self‐reported hearing difficulty was considered as a potential mediator in this study, the result showed that it indirectly mediated the association between residential greenness and tinnitus. However, the exact mechanisms of the greenness‐hearing difficulty‐tinnitus pathway remain unknown and need further investigation.

The interaction of our interest between greenness and MSRA gene polymorphism was also evaluated. In a stratified analysis according to the MSRA rs11249981 genotype, we observed that the association between greenness and tinnitus was most pronounced among the group with the homozygous T allele. The interaction of the MSRA gene polymorphism with the greenness was statistically significant. In two large independent cohorts from the UK Biobank and the Million Veterans the Million Veteran Program, Clifford et al.^[^ ^17^ ^]^ found a link between the MSRA gene SNP rs11249981 and tinnitus in a GWAS. Interestingly, previous studies have reported that the MSRA is a shared gene between depression and neuroticism,^[^ ^18^, ^22^ ^]^ as well as between response to selective serotonin reuptake inhibitors treatment and neuroticism.^[^ ^18^, ^21^ ^]^These indicated a possible genetic association of tinnitus with depression and neuroticism. Moreover, there is a high comorbidity between tinnitus and depression.^[^ ^1^, ^8^ ^]^ Neuroticism was strongly linked to current tinnitus.^[^ ^43^ ^]^ It has been established that residential greenness is associated with better mental health,^[^ ^24^, ^26^ ^]^ such as reduced risks for depression and anxiety. Similarly, there was a significant interaction of rs143424888 in the COL11A1 gene with greenness. A previous study reported a link between COL11A1 and Type II Stickler syndrome, which is associated with sensorineural hearing loss.^[^ ^44^ ^]^ In a GWAS by Clifford et al.,^[^ ^17^ ^]^ the effect size of rs143424888 in the COL11A1 gene was unchanged after further adjustment for hearing difficulty as a covariate, suggesting that this variant may be tinnitus‐specific. Moreover, we performed a sensitivity analysis to construct a PRS for tinnitus using multiple SNPs. A significant interaction between greenness and PRS was found. However, the selection of SNPs used to construct the PRS may be biased because these SNPs were obtained from the population in the UK Biobank cohort.^[^ ^45^ ^]^ In the future, large‐scale GWAS from independent populations are needed to calculate the PRS to validate our findings.

Several limitations of this study were also present. First, a longitudinal and negative association between greenness and tinnitus was replicated in a secondary analysis, but this was found in a relatively small and unbalanced sample. Further longitudinal studies with larger sample sizes are required to support our results. Second, greenness exposure measured at the participants' residences could lead to misclassification, while dynamic exposure in a mobility mode (e.g., workplace or other locations) was unavailable. Third, even though NDVI has considerable advantages as an objective indicator of greenness exposure, for example, the use of very high resolution (0.5 × 0.5 m^2^) enables very accurate measures of greenness, information on structure, quality, and type of vegetation cannot be distinguished by NDVI.^[^ ^26^ ^]^ Therefore, we could not assess what specific aspects of greenness were most relevant to tinnitus. Fourth, the study was unable to include detailed information on the characteristics of the tinnitus (e.g., perceptive position, loudness, pitch, onset, and duration) and whether or not to receive clinical help due to lack of them. Fifth, the SNPs selected for the construction of the PRS may be biased because they were not derived from a population independent of the UK Biobank cohort.^[^ ^45^ ^]^ More available and large‐scale GWASs from other populations are needed as a source of base data for calculating the PRS to test our findings of the significant interaction between greenness and genetic predisposition to tinnitus. Finally, residual confounding influences might remain despite adjustments for important variables.​

## 4 Conclusion

In summary, residential greenness was negatively associated with tinnitus in this large population‐based study. We also found a significant interaction between greenness and genetic predisposition to tinnitus. Indirect mediation of this association by hearing difficulty was also found. This study may be of great significance in developing tinnitus prevention or treatment strategies that are greenness‐related and cost‐effective, while helping to identify those who would benefit most. However, further longitudinal studies with larger sample sizes are required to validate our results and elucidate the exact mechanisms of the benefits of greenness for tinnitus.

## 5 Experimental Section

### Study Participants

UK Biobank was a large prospective cohort study with a wide range of health‐related information, including data from questionnaires (e.g., lifestyle and self‐reported tinnitus), physical measures (e.g., anthropometry), biological samples (including blood, urine, and saliva), imaging, and many different types of assays (including genetic, proteomic, and metabolomic analyses).​ During recruitment in 2006–2010, baseline assessment data was collected from ≈500000 participants aged 37–73 years at 22 assessment centers across the UK, with a subset of participants invited to take repeat baseline measures at multiple follow‐up visits. The ethical approval of UK Biobank was from the North West Multi‐center Research Ethics Committee (REC reference: 16/NW/0274). All participants provided an electronically signed consent form. More detailed information about UK Biobank can be found on its website (https://www.ukbiobank.ac.uk/).

In the UK Biobank baseline assessment, 172560 participants answered questions about tinnitus. After excluding participants with missing data for tinnitus, residential greenness, two SNPs (including rs11249981 and rs143424888), and variables at the individual level, a sample of 106471 participants was used for the primary (cross‐sectional) analyses (Figure S2, Supporting Information). Participants excluded tended to be younger and more female (Table S13, Supporting Information). At the 2012–2013 and 2014–2019 follow‐ups, the measures (including tinnitus) were updated for a subset of participants, respectively. A total of 15607 participants were included in the secondary (longitudinal) analysis, and a detailed selection of participants was provided in the Supplementary methods and Figure S3 (Supporting Information).

### Exposure Assessment

NDVI was used as an objective measure of residential greenness exposure for UK Biobank participants. NDVI was a unitless index calculated from spectral reflectance measurements of remotely sensed data, reflecting the relative overall greenness linked to vegetation cover. Comparing the radiation absorbed by the chlorophyll of healthy vegetation in the visible red region (630–690 nm) of the electromagnetic spectrum with that reflected in the near‐infrared region (760–900 nm), this ratio was used to estimate the greenness. The calculation formula is as follows:

(1) $$\mathrm{NDVI}=\frac{\;\left(\mathrm{NIR}-\mathrm{RED}\right)}{\;\left(\mathrm{NIR}+\mathrm{RED}\right)}$$

Where NIR and RED refer to spectral reflectance measurements obtained in the near‐infrared and visible (red) regions of the electromagnetic spectrum, respectively. NDVI ranges between −1 and 1, with higher values showing a higher density of healthy green vegetation. NDVI was obtained from the Blue Sky's Color Infrared imagery derived from specially developed sensors underneath a survey aircraft, which has a high resolution of 0.5 m × 0.5 m. To avoid potential temporal mismatches and effects of seasonal changes in greenness, summertime images of the study regions were collected on a similar time scale (during the baseline period of the UK Biobank study). According to previous studies,^[^ ^29^, ^46^, ^47^ ^]^ this study used the mean NDVI within a 0.5 km catchment radius based on the residential addresses of UK Biobank participants, following the exclusion of large water bodies.

### Outcome Assessment

At baseline, ≈170000 participants from the UK Biobank answered the self‐reported questions about tinnitus in a touch‐screen questionnaire. The question was, “Do you get or have you had noises (such as ringing or buzzing) in your head or in one or both ears that lasts for more than 5 min at a time?”. It is consistent with questions used in other major epidemiological studies of hearing, such as the Blue Mountains Hearing Study^[^ ^48^ ^]^ and the Epidemiology of Hearing Loss Study.^[^ ^49^ ^]^ In accordance with previous research on tinnitus,^[^ ^42^, ^43^ ^]^ current tinnitus in the primary analyses was defined as the responses of “Yes, now most or all of the time”, or “Yes, now a lot of the time”, or “Yes, now some of the time”. In a secondary analysis, incident tinnitus was defined as at least one report of tinnitus at subsequent follow‐up visits (2012–2013 and 2014–2019) among those who did not report current tinnitus at baseline, with the end of follow‐up occurring on December 31, 2019.

### Genotyping

Two candidate SNPs were chosen that were independently associated with tinnitus according to the GWAS by Clifford et al. in 2020:^[^ ^17^ ^]^ rs143424888 (COL11A1 gene) and rs11249981 (MSRA gene). More details about the genotyping process and arrays of the UK Biobank can be found elsewhere.^[^ ^50^ ^]^ Briefly, genotyping was performed using the Affymetrix UK Bi LEVE Axiom Array or the Affymetrix UK Biobank Axiom arrays, with imputation using two reference panels, including the Haplotype Reference Consortium data and the merged UK10 K and 1000 Genomes phase 3 panels. The Hardy‐Weinberg equilibrium (*P* values < 1.0 × 10^−6^) of the SNPs was examined using the genetics package in R. As a sensitivity analysis, the PRS was also calculated using the six SNPs (Table S14, Supporting Information) at the genome‐wide association significance levels identified by Clifford et al.^[^ ^17^ ^]^ A detailed description of the calculation of the PRS was found in the Supplementary methods.

### Confounders and Potential Mediators

According to prior knowledge and previous literature, the potential confounding variables were selected using a directed acyclic graph (DAG), which was a graphical tool to efficiently identify the confounders and quickly specify the minimal but sufficient adjustment set for minimizing confounding bias.^[^ ^51^ ^]^ With the help of DAGitty v3.0 software (www.dagitty.net), a minimally sufficient variables set adjusted for the association between residential greenness and tinnitus were (Figure S4, Supporting Information): sex (male, and female), age (in years), race (white, and non‐white), Townsend deprivation index (quartile), qualifications (college or university degree; A levels, AS levels or equivalent; O levels, general certificate of secondary educations, certificate of secondary educations or equivalent; national vocational qualification, higher national diploma, higher national certificate or other professional qualification; and none of the above), and employment (employed, retired, and other). The Townsend deprivation index, a proxy for socioeconomic status, was calculated from four variables on household overcrowding, non‐car ownership, non‐home ownership, and unemployment, which correspond to the area of each participant's postcode.^[^ ^52^ ^]^ The lower value of the Townsend deprivation index represents the higher socioeconomic status.

It has been reported that hearing loss was one of the leading risk factors for tinnitus.^[^ ^8^, ^9^ ^]^ Particularly, self‐reported hearing difficulty was strongly linked to tinnitus.^[^ ^42^, ^43^ ^]^ Furthermore, our previous study analyzed the association between residential greenness and hearing loss using data from the UK Biobank, suggesting a beneficial effect of greenness on lowering the risk of hearing loss. Therefore, it was hypothesized that hearing loss was on an intermediate pathway between residential greenness and tinnitus. Accordingly, self‐reported hearing difficulty was considered as a potential mediator added to the DAG (Figure S4, Supporting Information). Hearing difficulty (yes and no) was identified according to the touch‐screen question, “Do you have any difficulty with your hearing?”. In accordance with previous literature and the DAG, BMI, vascular problems, and diabetes were also treated as potential mediators. BMI was calculated by weight (in kilograms) divided by height (in meters) squared. In a touch‐screen questionnaire, vascular problems and diabetes were self‐reported. All confounders and potential mediators used in this study were measured at baseline.

### Statistical Analyses

Data were presented as mean (standard deviation) or median (IQR) for continuous variables and numbers (percentages) for categorical variables. Significant differences were calculated using the Wilcoxon rank sum test for continuous variables and the Chi‐square test for categorical variables.

In the primary (cross‐sectional) analysis, the association between residential greenness (NDVI) and tinnitus was assessed using logistic regression models with or without adjustment for confounding variables. NDVI was used as a continuous variable (an interquartile increment) and a five‐factor (quintile). The results were reported as ORs and corresponding 95% CIs. Two models were fitted: an unadjusted model, and an adjusted model with the inclusion of potential confounders selected from the DAG. Potential changes in the association of greenness with tinnitus stratified by the genotypes of rs11249981 and rs143424888 were also investigated. The cross‐product term for the counts of alleles of rs11249981 and rs143424888 and greenness were added to the logistic regression models to analyze the potential modification effects of the genes. For the primary analysis, an additional analysis and sensitivity analyses were also performed (Supplementary methods).

In the secondary analysis, the hazard ratio and the corresponding 95% CIs of the association between greenness at baseline and incident tinnitus were estimated using a Cox proportional hazard model with age as the time scale. Participants were considered at risk until the first diagnosis of tinnitus, death, loss to follow‐up, or end of follow‐up. The adjustment for covariates in the model was the same as used in the primary analysis.

Analyses were performed using R version 4.1.3. A two‐tailed *P* value <0.05 was used as the level of statistical significance.

## Conflict of Interest

The authors declare no conflict of interest.

## Supporting information

Supporting Information

## Data Availability

Data supporting reported results can be found from the website of UK Biobank (www.ukbiobank.ac.uk) upon application.
