# Supplementary material for: Greenness, Genetic Predisposition, and Tinnitus
Source: Adv Sci (Weinh). 2024 Mar 6;11(17):2306706. doi: 10.1002/advs.202306706 (PMC11077638; doi:10.1002/advs.202306706)
Supplement: Supplementary file 1 — Supporting Information [file ADVS-11-2306706-s001.pdf]

ADVANCED SCIENCE
Open Access

Supporting Information

for *Adv. Sci.*, DOI 10.1002/advs.202306706

Greenness, Genetic Predisposition, and Tinnitus

*Lan-Lai Yuan, Dan-Kang Li, Yao-Hua Tian and Yu Sun**

Supporting Information

Greenness, Genetic Predisposition, and Tinnitus

*Lan-Lai Yuan, Dan-Kang Li, Yao-Hua Tian, Yu Sun**

L.-L. Yuan

Department of Otorhinolaryngology, Union Hospital, Tongji Medical College, Huazhong University of Science and Technology, Wuhan, 430022, China;

D.-K. Li, Y.-H. Tian

Ministry of Education Key Laboratory of Environment and Health, and State Key Laboratory of Environmental Health (Incubating), School of Public Health, Tongji Medical College, Huazhong University of Science and Technology, Wuhan 430022, China; Department of Maternal and Child Health, School of Public Health, Tongji Medical College, Huazhong University of Science and Technology, Wuhan 430022, China;

Y. Sun

Department of Otorhinolaryngology, Union Hospital, Tongji Medical College, Huazhong University of Science and Technology, Wuhan 430022, China; Institute of Otorhinolaryngology, Union Hospital, Tongji Medical College, Huazhong University of Science and Technology Wuhan 430022, China; Hubei Province Key Laboratory of Oral and Maxillofacial Development and Regeneration, Wuhan 430022, China.

Email: sunyu@hust.edu.cn.

Supplementary methods

Selection of participants in the secondary (longitudinal) analysis

Of the 172560 participants at baseline, we excluded participants with prevalent tinnitus (n = 29852), those with missing data on tinnitus at baseline (n = 4278), those who died at baseline (n = 169), those with missing data on tinnitus at both follow-ups in 2012 – 2013 and 2014 – 2019 (n = 117195), and those with missing data on normalized difference vegetation index (NDVI) (n = 3719) and covariates at baseline (n = 1740). As a result, a total of 15607 participants were included in the secondary analysis.

Calculation of polygenic risk score

We used six single nucleotide polymorphisms (SNPs) at genome-wide significance levels for tinnitus identified by Clifford et al. [1] in the European population of the UK Biobank cohort to calculate the polygenic risk score (PRS). The weighted PRS for tinnitus was calculated using the method described in a previous study [2] with the following formula: PRS = ($\beta 1 \times SNP_1 + \beta_2 \times SNP_2 + \beta_3 \times SNP_3 + \beta_4 \times SNP_4 + \beta_5 \times SNP_5 + \beta_6 \times SNP_6$), where β_i (i = 1, 2, ..., 6) was the estimated risk for each SNP in the genome-wide association study, and SNP_i (i = 1, 2, ..., 6) was the number of risk alleles for each SNP. Participants were divided into three groups according to the PRS of tinnitus: low (tertile 1), intermediate (tertile 2), and high (tertile 3).

Statistical analyses for primary analyses

To test for the non-linearity of association, we also used a restricted cubic spline model with Harrel knots. Moreover, we employed the PROCESS function in the R package bruceR for analysis with 1000 bootstrap samples to assess the effects of potential mediators on the association between greenness and tinnitus. The percentages of the mediation effects was calculated by: (indirect effect / total effect) × 100%.

We also conducted several sensitivity analyses. First, we examined the interaction between greenness and PRS by using the cross-product term of NDVI and PRS in the logistic regression model. Additionally, the multiplicative interactions of each reported SNP with greenness were examined separately. To assess the joint effect of greenness and PRS, we constructed a joint category according to the quintiles of NDVI and the tertiles of PRS. The genotyping batch and the first 10 genetic principal components were further adjusted based on the primary analysis. Second, average total income before tax at household level was additionally adjusted in the main model. Household income was coded as a four-factor variable (<£18000, £18000-30999, £31000-51999 and ≥£52000). Third, repeated analyses were performed by additional adjustments for smoking, drinking frequency, occupational as well as musical noise exposure, and all of the above to reduce potential residual confounders. Fourth, only those who did not use hearing aids were included to reduce the possible impact of hearing aids. Fifth, we excluded participants who had lived at their current address for less than three years to reduce the influence of migration. Sixth, we

examined the effect modification of the occurrence of one or more of the following five comorbidities: hypertension, heart disease, angina, stroke, and diabetes. Finally, to remove the effect of the history of tinnitus, we redefined the group without tinnitus by excluding participants who had reported tinnitus in the past.

References

1. Clifford R E, Maihofer A X, Stein M B, et al. Novel Risk Loci in Tinnitus and Causal Inference With Neuropsychiatric Disorders Among Adults of European Ancestry [J]. JAMA Otolaryngol Head Neck Surg, 2020, 146(11): 1015-25. https://doi.org/10.1001/jamaoto.2020.2920
2. Wang N, Yu Y, Sun Y, et al. Acquired risk factors and incident atrial fibrillation according to age and genetic predisposition [J]. European Heart Journal, 2023, 44(47): 4982-93. https://doi.org/10.1093/eurheartj/ehad615

Supplementary figures

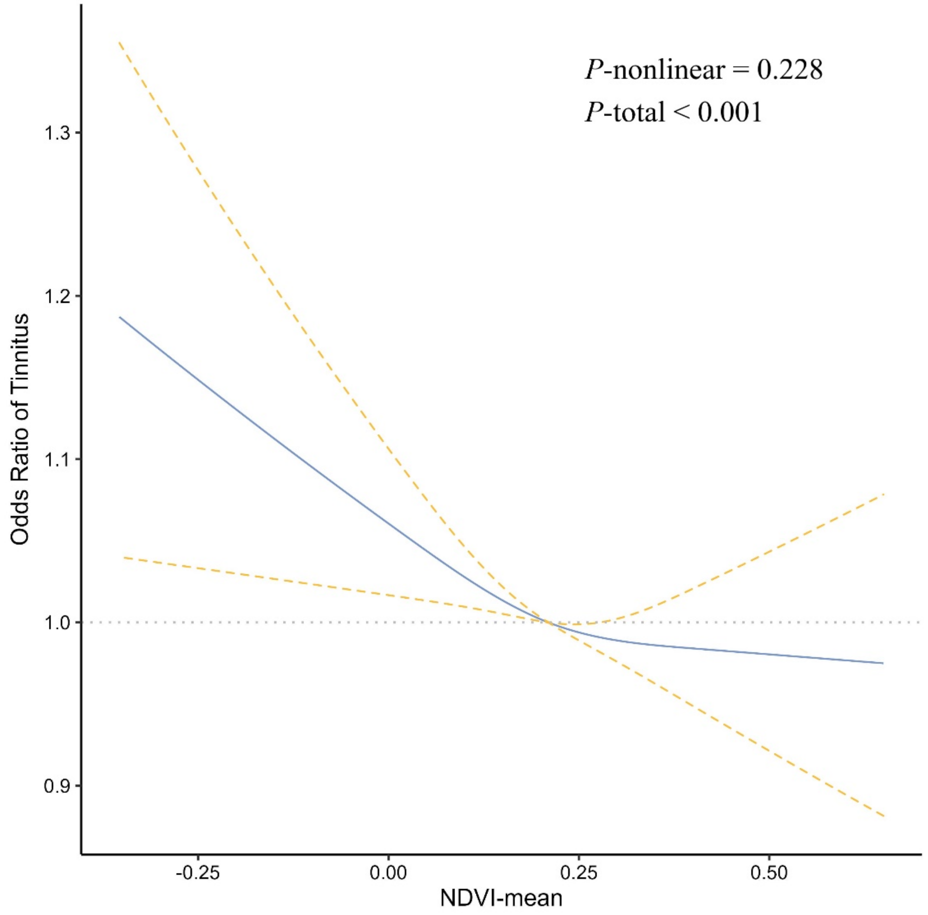

Figure S1. Dose-response curves for greenness with tinnitus among 106471 participants.

Abbreviations: NDVI, normalized difference vegetation index.
A restricted cubic spline model with three knots placed at 10th, 50th, and 90th percentiles was used (*P* for nonlinear association = 0.228). The dotted lines represent 95% confidence intervals.

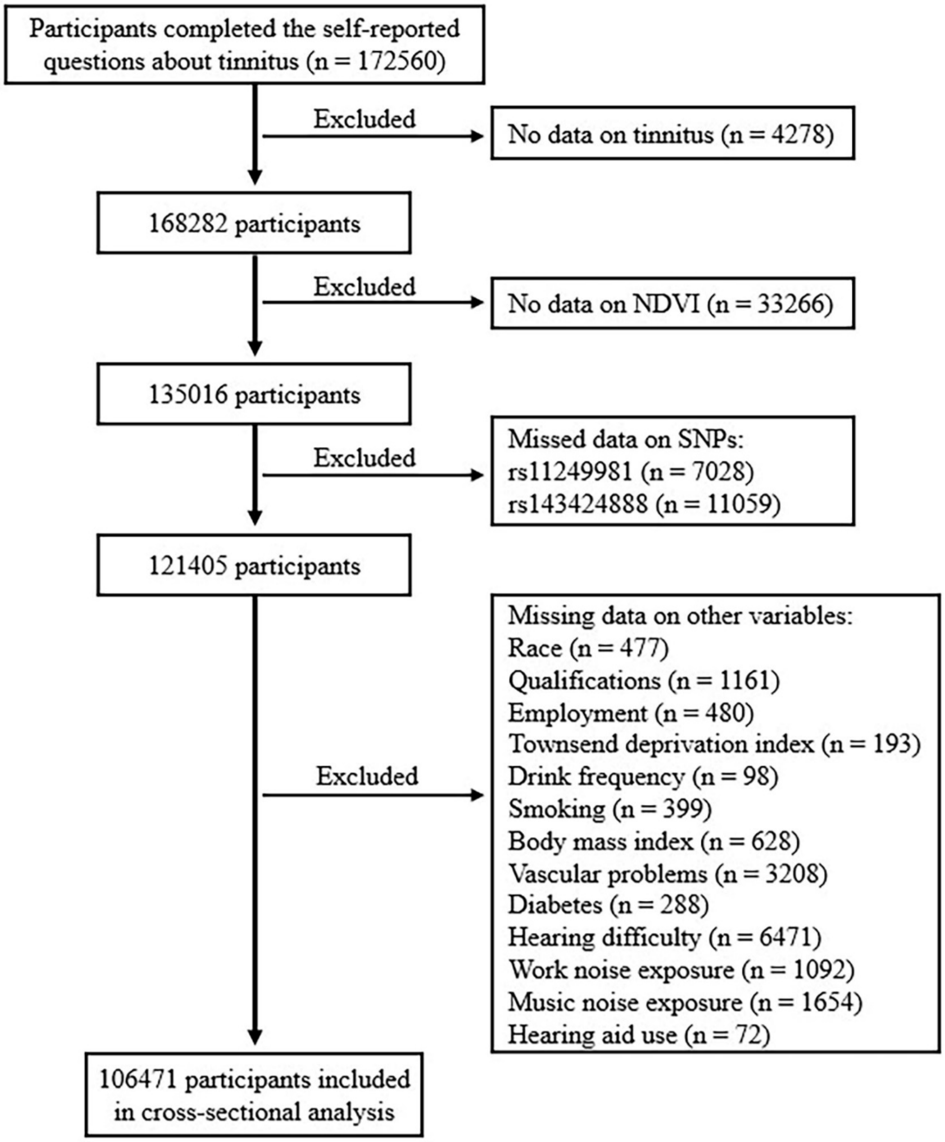

Figure S2. Flow chart for cross-sectional analyses in this study from the UK Biobank cohort between 2006 and 2010.

Abbreviations: NDVI, normalized difference vegetation index; SNPs, single nucleotide polymorphisms.

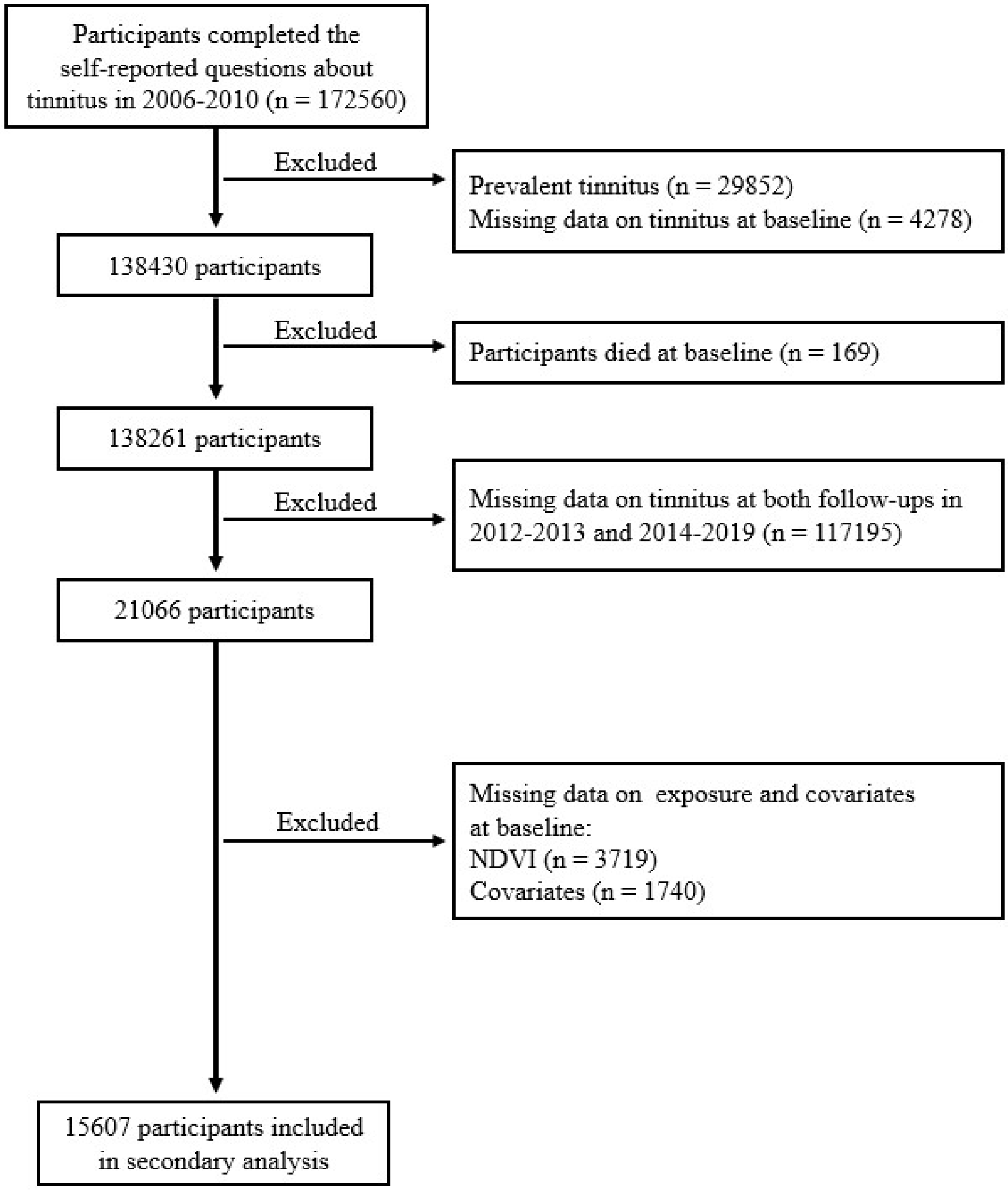

Figure S3. Flow chart for secondary analysis in this study.

Abbreviations: NDVI, normalized difference vegetation index.

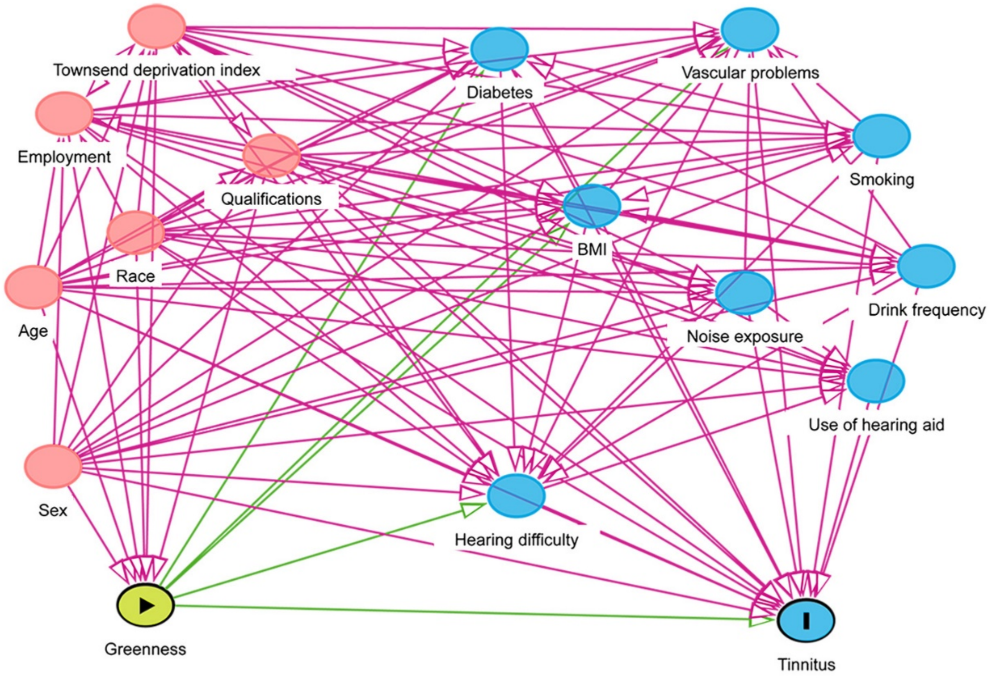

Figure S4. Directed acyclic graph for the association between greenness and tinnitus.

The directed acyclic graph was created with the help of DAGitty.net (www.dagitty.net). Pink lines indicate potential confounders and green lines indicate potential mediators. Minimally sufficient adjustment set: age, sex, race, Townsend deprivation index, qualifications, employment.

Supplementary tables

Table S1. Cross-sectional association between residential greenness and the odds of tinnitus stratified by the genotype of rs11249981 and rs143424888 (n = 106471).

SNP	Number of participants	OR[a)] per IQR increase	*P*	*P* - interaction
rs11249981				0.024
C/C	21741	1.02 (0.97,1.07)	0.521	
C/T	52780	0.96 (0.93,0.99)	0.015	
T/T	31950	0.94 (0.91,0.98)	0.004	
rs143424888				0.042
C/C	28742	0.94 (0.90,0.98)	0.004	
C/Ins[b)]	52460	0.97 (0.94,1.00)	0.041	
Ins[b)]/Ins[b)]	25269	1.00 (0.95,1.04)	0.835	

Abbreviations: SNP, single nucleotide polymorphism; OR, odds ratio; IQR, interquartile range.

Data presented as odds ratio (95% CI).

[a)]Adjusted for age, sex, race, Townsend deprivation index, qualifications, and employment.

[b] Insertion of CACGTGATCT.

Table S2. Cross-sectional association[a)] between quintile greenness and the odds of tinnitus stratified by the genotype of rs11249981 (n = 106471).

SNP	Quintiles of NDVI					OR per one quintile change in NDVI[b)]	*P*-trend[c)]
	Q1	Q2	Q3	Q4	Q5		
rs11249981							
C/C	1.00 (ref)	0.95 (0.85,1.07)	0.98 (0.87,1.10)	0.99 (0.88,1.11)	1.02 (0.92,1.15)	1.01 (0.98,1.04)	0.478
C/T	1.00 (ref)	1.01 (0.94,1.08)	0.97 (0.90,1.04)	0.96 (0.89,1.03)	0.94 (0.87,1.01)	0.98 (0.97,1.00)	0.031
T/T	1.00 (ref)	0.93 (0.85,1.02)	0.91 (0.83,0.99)	0.90 (0.82,0.99)	0.91 (0.83,0.99)	0.98 (0.96,1.00)	0.035

Abbreviations: SNP, single nucleotide polymorphism; NDVI, normalized difference vegetation index; OR, odds ratio; Q1, Q2, Q3, Q4 and Q5: 1st (-0.4964 – 0.0816, n = 21291), 2nd (0.0817 – 0.1848, n = 21278), 3rd (0.1849 – 0.2417, n = 21286), 4th (0.2418 – 0.3253, n = 21303), and 5th (0.3254 – 0.7032, n = 21313) quintiles of NDVI respectively.

Data presented as odds ratio (95% CI).

[a)]Adjusted for age, sex, race, Townsend deprivation index, qualifications, and employment.

[b)] Odds ratio are presented per quintile increase in NDVI by the genotype of rs11249981.

[c)]*P*-trend describes the association between residential greenness and the odds of tinnitus within quintiles for NDVI.

Table S3. Cross-sectional association[a)] between residential greenness, potential mediators, and tinnitus (n = 106471).

Mediators		Effects (SE)	(95% CI)[b)]	*P*
Hearing difficulty				
	Indirect	-0.009 (0.002)	(-0.013, -0.005)	<0.001
	Direct	-0.016 (0.008)	(-0.030, 0.000)	0.046
	Total	-0.024 (0.008)	(-0.040, -0.008)	0.002
BMI				
	Indirect	-0.000 (0.000)	(-0.001, -0.000)	0.009
	Direct	-0.024 (0.008)	(-0.039, -0.007)	0.003
	Total	-0.024 (0.008)	(-0.039, -0.007)	0.002
Vascular problems[c)]				
	Indirect	-0.000 (0.000)	(-0.001, -0.000)	0.030
	Direct	-0.024 (0.008)	(-0.039, -0.007)	0.003
	Total	-0.024 (0.008)	(-0.039, -0.007)	0.002
Diabetes				
	Indirect	0.000 (0.000)	(-0.001, -0.000)	0.672
	Direct	-0.032 (0.009)	(-0.049, -0.015)	<0.001
	Total	-0.032 (0.009)	(-0.049, -0.015)	<0.001

Abbreviations: SE, standard error; CI, confidence interval; BMI, body mass index.

[a)] Adjusted for age, sex, race, Townsend deprivation index, qualifications, and employment.

[b)] Confidence interval was estimated by bias-corrected and accelerated percentile bootstrap.

[c)] Vascular problems were treated as a dichotomous variable: yes (including hypertension, and heart attack, angina, or stroke) and no.

SE and CI are estimated based on 1000 Bootstrap samples

Table S4. The joint association of the residential greenness (in quintiles) and polygenic risk score with the risk of tinnitus (n = 99689).

Polygenic risk score	NDVI-quintile	Number of participants	Adjusted[a)] OR (95% CI)	*P*
High genetic risk				
	Q1	6610	1.00 (ref)	
	Q2	6401	0.96 (0.88, 1.05)	0.416
	Q3	7524	0.93 (0.86, 1.02)	0.123
	Q4	7255	0.93 (0.86, 1.02)	0.135
	Q5	7069	0.89 (0.82, 0.97)	0.011
Intermediate genetic risk				
	Q1	6498	0.95 (0.87, 1.04)	0.271
	Q2	6170	0.93 (0.85, 1.02)	0.104
	Q3	7511	0.86 (0.79, 0.94)	<0.001
	Q4	7735	0.84 (0.77, 0.92)	<0.001
	Q5	6889	0.87 (0.79, 0.95)	0.002
Low genetic risk				
	Q1	5627	0.88 (0.80, 0.97)	0.009
	Q2	5366	0.81 (0.73, 0.89)	<0.001
	Q3	6376	0.82 (0.75, 0.90)	<0.001
	Q4	6640	0.92 (0.84, 1.01)	0.066
	Q5	6018	0.89 (0.81, 0.98)	0.014

Abbreviations: OR, odds ratio; CI, confidence interval; NDVI, normalized difference vegetation index; Q1, Q2, Q3, Q4 and Q5: 1st (-0.4964 – 0.0750, n = 18735), 2nd (0.0751 – 0.1750, n = 17937), 3rd (0.1751 – 0.2350, n = 21411), 4th (0.2351 – 0.3250, n = 21630), and 5th (0.3251 – 0.7032, n = 19976) quintiles of greenness respectively. The *P* value of cross-product interaction between NDVI and polygenic risk score was 0.004.

[a)] Adjusted for age, sex, race, Townsend deprivation index, qualifications, employment, genotyping batch, and the first ten genetic principal components.

Table S5. Association between polygenic risk score and the odds of tinnitus (n = 99689).

Polygenic risk score	Number of participants	Adjusted[a)] OR (95% CI)	*P*
Continuous		3.67 (2.41, 5.60)	<0.001
Low genetic risk	30027	1.00 (ref)	
Intermediate genetic risk	34803	1.02 (0.98, 1.07)	0.255
High genetic risk	34859	1.09 (1.05, 1.14)	<0.001
P-trend[b)]			<0.001

Abbreviations: OR, odds ratio; CI, confidence interval

[a)] Adjusted for age, sex, race, Townsend deprivation index, qualifications, employment, genotyping batch, and the first ten genetic principal components.

[b)] *P*-trend describes the association between polygenic risk score and tinnitus within three groups for polygenic risk score.

Table S6. Cross-sectional association between residential greenness and the odds of tinnitus (n = 99689).

	Adjusted[a)] OR (95% CI)	*P*
NDVI, per IQR	0.96 (0.94, 0.99)	0.002
NDVI-quintile		
Q1	1.00 (ref)	
Q2	0.97 (0.91, 1.02)	0.207
Q3	0.95 (0.90, 1.00)	0.060
Q4	0.95 (0.90, 1.00)	0.059
Q5	0.94 (0.89, 0.99)	0.019
P-trend[b)]		0.019

Abbreviations: OR, odds ratio; CI, confidence interval; NDVI, normalized difference vegetation index; IQR, interquartile range; Q1, Q2, Q3, Q4 and Q5: 1st (-0.4964 – 0.0750, n = 18735), 2nd (0.0751 – 0.1750, n = 17937), 3rd (0.1751 – 0.2350, n = 21411), 4th (0.2351 – 0.3250, n = 21630), and 5th (0.3251 – 0.7032, n = 19976) quintiles of greenness respectively.

The NDVI of each IQR is equivalent to 0.19.

a) Adjusted for age, sex, race, Townsend deprivation index, qualifications, and employment.

b) *P*-trend describes the association between greenness and tinnitus within quintiles for NDVI.

Table S7. The associations between residential greenness and odds of tinnitus stratified by each single nucleotide polymorphism (n = 99689).

**SNPs**	Number of participants	OR[a)] per IQR increase	*P* for interaction
rs143424888			0.019
C/C	26965	0.94 (0.90, 0.98)	
C/Ins[b)]	49061	0.96 (0.93, 0.99)	
Ins[b)]/Ins[b)]	23663	1.00 (0.96, 1.05)	
rs11249981			0.015
C/C	20373	1.01 (0.96, 1.07)	
C/T	49452	0.96 (0.93, 0.996)	
T/T	29864	0.93 (0.90, 0.97)	
rs553448379			0.725
T/T	16534	0.95 (0.90, 1.00)	
T/TA	46219	0.98 (0.95, 1.02)	
TA/TA	36936	0.95 (0.92, 0.98)	
rs11174489			0.161
G/G	38982	0.97 (0.94, 1.01)	
G/A	46740	0.97 (0.94, 1.00)	
A/A	13967	0.92 (0.87, 0.98)	
rs17249745[c)]			0.254
A/A	95083	0.96 (0.94, 0.98)	
A/G	4548	1.02 (0.92, 1.13)	
G/G	58	-	
A/G + G/G	4606	1.02 (0.92, 1.13)	
rs72815660[c)]			0.068
A/A	92182	0.97 (0.95, 0.99)	
A/G	7352	0.91 (0.84, 0.98)	
G/G	155	-	
A/G + G/G	7507	0.90 (0.83, 0.98)	

Abbreviations: SNPs, single nucleotide polymorphisms; OR, odds ratio; IQR, interquartile range.

Data presented as odds ratio (95% CI).

[a] Adjusted for age, sex, race, Townsend deprivation index, qualifications, and employment.

[b] Insertion of CACGTGATCT.

[c] The G/G genotype of rs17249745 and rs72815660 were merged with A/G due to the small number of participants with the G/G genotype.

Table S8. Cross-sectional association between residential greenness and the odds of tinnitus with additional adjustment for household income (n = 93353).

	Adjusted[a)] OR (95% CI)	*P*
NDVI, per IQR	0.97 (0.95,0.99)	0.007
NDVI-quintile		
Q1	1.00 (ref)	
Q2	0.95 (0.90, 1.01)	0.077
Q3	0.94 (0.89, 0.99)	0.030
Q4	0.95 (0.90, 1.01)	0.086
Q5	0.94 (0.89, 0.99)	0.017
P-trend[b)]		0.033

Data presented as odds ratio (95% CI).

Abbreviations: OR, odds ratio; CI, confidence interval; NDVI, normalized difference vegetation index; IQR, interquartile range; Q1, Q2, Q3, Q4 and Q5: 1st (-0.4964 – 0.0817, n = 18658), 2nd (0.0818 – 0.1851, n = 18661), 3rd (0.1852 – 0.2416, n = 18675), 4th (0.2417 – 0.3247, n = 18680), and 5th (0.3248 – 0.7032, n = 18679) quintiles of greenness respectively.

The NDVI of each IQR is equivalent to 0.19.

[a)] Adjusted for age, sex, race, Townsend deprivation index, qualifications, employment, and household income.

[b)] *P*-trend describes the association between greenness and tinnitus within quintiles for NDVI.

Table S9. Cross-sectional association between residential greenness and the odds of tinnitus after additional adjustments for smoking, drink frequency, occupational and musical noise exposure, and all of them (n = 106471).

	OR per IQR increase	*P*
Main model[a)] + smoking	0.97 (0.95,0.99)	0.002
Main model[a)] + drink frequency	0.97 (0.95,0.99)	0.005
Main model[a)] + occupational noise exposure	0.97 (0.94,0.99)	0.002
Main model[a)] + musical noise exposure	0.97 (0.95,0.99)	0.005
Main model[a)] + all of the above	0.97 (0.95,0.99)	0.006

Data presented as odds ratio (95% CI).

Abbreviations: NDVI, normalized difference vegetation index; OR, odds ratio; IQR, interquartile range.

[a)]Adjusted for age, sex, race, Townsend deprivation index, qualifications, and employment.

Table S10. Cross-sectional association[a)] between residential greenness and the odds of tinnitus after excluding participants who had used hearing aids and had lived in their current residence for less than three years.

	Number of participants	OR per IQR increase	*P*
Without use of hearing aids	103247	0.97 (0.94,0.99)	0.003
≥ 3 years in current address	98481	0.97 (0.94,0.99)	0.004

Data presented as odds ratio (95% CI).

Abbreviations: NDVI, normalized difference vegetation index; OR, odds ratio; IQR, interquartile range.

[a)]Adjusted for age, sex, race, Townsend deprivation index, qualifications, and employment.

Table S11. Cross-sectional association[a] between residential greenness and the odds of tinnitus stratified by comorbidities (n = 106471).

	Number of participants	OR per IQR increase	*P*	*P* - interaction
Comorbidities[b]				0.782
Yes	31066	0.97 (0.93,1.01)	0.103	
No	75405	0.97 (0.94,0.99)	0.013	

Abbreviations: OR, odds ratio; IQR, interquartile range.

Data presented as odds ratio (95% CI).

[a]Adjusted for age, sex, race, Townsend deprivation index, qualifications, and employment.

[b] Comorbidities included hypertension, heart disease, angina, stroke, and diabetes, with participants who had none of the above being defined as controls.

Table S12. Cross-sectional association between residential greenness and the odds of the alternative definition of tinnitus (n = 94978).

	Adjusted[a)] OR (95% CI)	*P*
NDVI, per IQR	0.96 (0.94,0.98)	< 0.001
NDVI-quintile		
Q1	1.00 (ref)	
Q2	0.97 (0.93,1.02)	0.311
Q3	0.93 (0.89,0.98)	0.011
Q4	0.93 (0.89,0.98)	0.011
Q5	0.93 (0.89,0.98)	0.009
P-trend[b)]		0.003

Abbreviations: OR, odds ratio; CI, confidence interval; NDVI, normalized difference vegetation index; IQR, interquartile range; Q1, Q2, Q3, Q4 and Q5: 1st (-0.4964 – 0.0821, n = 18986), 2nd (0.0822 – 0.1852, n = 18998), 3rd (0.1853 – 0.2421, n = 18996), 4th (0.2422 – 0.3254, n = 18985), and 5th (0.3255 – 0.7032, n = 19013) quintiles of greenness respectively.

The NDVI of each IQR change is equivalent to 0.19.

[a)]Adjusted for age, sex, race, Townsend deprivation index, qualifications, and employment.

[b)]*P*-trend describes the association between residential greenness and the alternative definition of tinnitus within quintiles for NDVI.

Table S13. Characteristics of participants who were and were not included in the primary analysis.

	Excluded (n = 66089)	Included (n = 106471)
**Age (years), median (IQR)**	58.0 (13.0)	61.0 (11.0)
**NDVI, median (IQR)**	0.22 (0.18)	0.21 (0.19)
**Sex (%)**		
Female	36507 (55.2)	57362 (53.9)
Male	29582 (44.8)	49109 (46.1)
**Race (%)**		
White	56443 (86.5)	100155 (94.1)
Non-white	8797 (13.5)	6316 (5.9)
**Qualifications (%)**		
College or university degree	20140 (31.5)	37543 (35.3)
A levels or A levels or equivalent	7041 (11.0)	12389 (11.6)
O levels, GCEs, or CEs or equivalent	17510 (27.3)	28827 (27.1)
NVQ, HND, HNC, or other professional qualification	7789 (12.2)	12623 (11.9)
None of the above	11548 (18.0)	15089 (14.2)
**Employment (%)**		
Employed	35410 (54.3)	60049 (56.4)
Retired	22349 (34.3)	37090 (34.8)
Other	7426 (11.4)	9332 (8.8)
**Townsend deprivation index (%)**		
1st (least deprived)	16451 (25.0)	26599 (25.0)
2nd	16451 (25.0)	26627 (25.0)
3rd	16452 (25.0)	26626 (25.0)
4th (most deprived)	16452 (25.0)	26619 (25.0)
**Drink frequency (%)**		
Daily or almost daily	11828 (18.0)	22920 (21.5)
Three or four times a week	13269 (20.2)	24849 (23.3)
Once or twice a week	16129 (24.5)	27036 (25.4)
One to three times a month	7700 (11.7)	11841 (11.1)
pecial occasions only	9415 (14.3)	11828 (11.1)
Never	7464 (11.3)	7997 (7.5)
**moking (%)**		
Never	36065 (55.2)	58910 (55.3)
Previous	22019 (33.7)	37326 (35.1)
Current	7204 (11.0)	10235 (9.6)
**BMI, kg/m² (%)**		
<25	20365 (31.5)	36402 (34.2)
≥25 and <30	26965 (41.7)	45204 (42.5)
≥30	17400 (26.9)	24865 (23.4)
**Diabetes (%)**		
No	60849 (93.1)	100995 (94.9)
Yes	4475 (6.9)	5476 (5.1)
**Vascular problems (%)**		
None	44056 (71.9)	77415 (72.7)

Hypertension	13854 (22.6)	23580 (22.1)
Heart attack, angina, or stroke	1655 (2.7)	2670 (2.5)
Hypertension, and heart attack, angina, or stroke	1716 (2.8)	2806 (2.6)
**Hearing difficulty (%)**		
No	40599 (72.3)	77360 (72.7)
Yes	15562 (27.7)	29111 (27.3)
**Hearing aid use (%)**		
No	63674 (97.0)	103247 (97.0)
Yes	1981 (3.0)	3224 (3.0)
**Work noise exposure (%)**		
No	48441 (75.9)	82,491 (77.5)
Yes	15411 (24.1)	23,980 (22.5)
**Music noise exposure (%)**		
No	54706 (86.8)	93698 (88.0)
Yes	8297 (13.2)	12773 (12.0)

Abbreviations: IQR, interquartile range; NDVI, normalized difference vegetation index; GCEs, general certificate of secondary educations; CEs, certificate of secondary educations; NVQ, national vocational qualification; HND, higher national diploma; HNC, higher national certificate; BMI, body mass index.

Data are shown as means (standard deviation) or medians (IQR) for continuous variables and numbers (percentages) for categorical variables.

Table S14. Summary information of six single nucleotide polymorphisms used for polygenic risk score in this study.

SNP	Chromosome	Position[a]	Beta	SE	*P* value	Allele 1	Allele 2	Allele 1 frequency
rs143424888	1	103456996	0.023	0.004	8.50E-09	C	Ins[b]	0.510
rs553448379	6	43288656	-0.023	0.004	2.10E-08	T	TA	0.386
rs11249981	8	10147398	-0.022	0.004	4.60E-08	C	T	0.447
rs11174489	12	62852271	-0.024	0.004	3.80E-09	G	A	0.625
rs17249745	4	102547366	-0.072	0.012	1.3E-09	A	G	0.967
rs72815660	10	106614698	-0.060	0.010	9.70E-09	A	G	0.960

Abbreviations: SNP, single nucleotide polymorphism; SE, standard error; Ins, insertion variant.

[a] Indicates base pair position on chromosome (GR37 Human Genome Build hg19).

[b] Insertion of CACGTGATCT.
